# Oxytocin Promotes Accurate Fear Discrimination and Adaptive Defensive Behaviors

**DOI:** 10.3389/fnins.2020.583878

**Published:** 2020-09-23

**Authors:** Valentina Olivera-Pasilio, Joanna Dabrowska

**Affiliations:** ^1^Center for the Neurobiology of Stress Resilience and Psychiatric Disorders, Rosalind Franklin University of Medicine and Science, North Chicago, IL, United States; ^2^Discipline of Cellular and Molecular Pharmacology, Chicago Medical School, Rosalind Franklin University of Medicine and Science, North Chicago, IL, United States; ^3^School of Graduate and Postdoctoral Studies, Rosalind Franklin University of Medicine and Science, North Chicago, IL, United States

**Keywords:** oxytocin, amygdala, BNST, cued fear, contextual fear, fear discrimination, social fear, social transmission of fear

## Abstract

The nonapeptide, oxytocin (OT), known for its role in social bonding and attachment formation, has demonstrated anxiolytic properties in animal models and human studies. However, its role in the regulation of fear responses appears more complex, brain site-specific, sex-specific, and dependent on a prior stress history. Studies have shown that OT neurons in the hypothalamus are activated during cued and contextual fear conditioning and during fear recall, highlighting the recruitment of endogenous oxytocin system in fear learning. OT is released into the extended amygdala, which contains the central nucleus of the amygdala (CeA) and the bed nucleus of the stria terminalis (BNST), both critical for the regulation of fear and anxiety-like behaviors. Behavioral studies report that OT in the CeA reduces contextual fear responses; whereas in the BNST, OT receptor (OTR) neurotransmission facilitates cued fear and reduces fear responses to un-signaled, diffuse threats. These ostensibly contrasting behavioral effects support growing evidence that OT works to promote fear discrimination by reducing contextual fear or fear of diffuse threats, yet strengthening fear responses to imminent and predictable threats. Recent studies from the basolateral nucleus of the amygdala (BLA) support this notion and show that activation of OTR in the BLA facilitates fear discrimination by increasing fear responses to discrete cues. Also, OTR transmission in the CeA has been shown to mediate a switch from passive freezing to active escape behaviors in confrontation with an imminent, yet escapable threat but reduce reactivity to distant threats. Therefore, OT appears to increase the salience of relevant threat-signaling cues yet reduce fear responses to un-signaled, distant, or diffuse threats. Lastly, OTR signaling has been shown to underlie emotional discrimination between conspecifics during time of distress, social transmission of fear, and social buffering of fear. As OT has been shown to enhance salience of both positive and negative social experiences, it can also serve as a warning system against potential threats in social networks. Here, we extend the social salience hypothesis by proposing that OT enhances the salience of relevant environmental cues also in non-social contexts, and as such promotes active defensive behaviors.

## Introduction

The nonapeptide, oxytocin (OT), produced in the paraventricular (PVN), supraoptic (SON), and accessory nuclei (AN) of the hypothalamus, is released to and then secreted from the posterior pituitary gland to the peripheral blood circulation (Sofroniew, 1983; Swanson and Sawchenko, 1983). After reaching the target organs, OT regulates important aspects of reproductive behavior such as uterus contractions during parturition and milk ejection during lactation (Nickerson et al., 1954; Caldeyro-Barcia and Poseiro, 1959; for review see Afzal et al., 2017). In addition, axon collaterals from the OT neurons in the hypothalamus send dense OT projections into the central nervous system (CNS), where OT plays an important role in the regulation of affective and social behaviors (Neumann, 2007; Knobloch et al., 2012; Stoop, 2012; Bosch and Young, 2017; Jurek and Neumann, 2018). OT binds to oxytocin receptors (OTR), which belong to the G-protein coupled receptor (GPCR) family and demonstrate high expression levels in brain regions implicated in emotional regulation (Brinton et al., 1984; Tribollet et al., 1988; Veinante and Freund-Mercier, 1997; for review see Busnelli and Chini, 2017; Jurek and Neumann, 2018).

OT has been shown to modulate stress response (Neumann et al., 2000; Olff et al., 2013), stress-coping mechanisms and has demonstrated anxiolytic and anti-depressant-like effects in animal models (Bale et al., 2001; Ring et al., 2006; Missig et al., 2010; Wang T. et al., 2018; for review see Janeček and Dabrowska, 2019). However, the role of OT in the regulation of fear appears to be more complex (Lahoud and Maroun, 2013; Campbell-Smith et al., 2015). The extended amygdala, particularly important for the regulation of fear memory (Veinante and Freund-Mercier, 1997; Jurek and Neumann, 2018), has high levels of OTR binding sites and OTR expression. Notably, forebrain OTRs have been shown essential for fear learning in male mice (Pagani et al., 2011). Transgenic OTR knock-out (KO) mice restricted to the forebrain showed an attenuation of freezing responses during fear acquisition and during both cued and contextual fear recall. Interestingly, brain-wide OTR KO mice did not significantly differ from their wild-type counterparts in their fear expression levels (Pagani et al., 2011).

Fear is a combination of behavioral (e.g., freezing, vigilance, startle) and physiological (e.g., cardiovascular, respiratory) responses experienced during an exposure to an actual or potential threat that can compromise survival (Gross and Canteras, 2012; Tovote et al., 2015; for reviews see LeDoux, 2000, 2014; Asok et al., 2019). Although rodents likely do not experience fear the way humans do (LeDoux and Pine, 2016), what makes fear response so well conserved among species (e.g., rats, monkeys, and humans) is the fact that it stimulates autonomic responses to a potential danger (e.g., cardiovascular) and potentiates reflexes (e.g., startle). Because of the autonomic and reflexive nature of these responses, these are impossible to control, even by a conscious brain. Although some fear responses are innate (fear of falling, fear of loud noises, fear of predators) (Blanchard et al., 1990; Hogg and File, 1994) for review see Gross and Canteras (2012), the great majority of fear responses are learned from experience (Olsson and Phelps, 2004; Adolphs, 2013; LeDoux, 2014).

In Pavlovian fear conditioning, a neutral sensory stimulus (usually a tone or light) is paired with an aversive event such as a foot-shock (unconditioned stimulus, US). After presentations of the pairings, the neutral stimulus acquires fear-eliciting properties and hence becomes the conditioned stimulus (CS). During the fear recall test, the CS triggers an array of physiological and behavioral responses resembling a fear response when presented alone (Gross and Canteras, 2012; Tovote et al., 2015). In contextual fear learning, subjects learn to associate a neutral context with an aversive event, which results in eliciting fear responses after re-exposure to that context in the absence of any threat. The most common behavioral outcomes of fear response measured in the laboratory conditions are freezing behavior and potentiation of an acoustic startle reflex. These forms of associative learning can be divided into several phases of memory formation including acquisition (training, fear-conditioning), consolidation, recall, extinction, and retrieval or reinstatement of the learned response (Maren, 2001; Haaker et al., 2019).

The amygdala complex, which consists of the lateral amygdala (LA) and the basolateral nucleus of the amygdala (BLA), is critical for the acquisition and storage of fear memories (Fanselow and LeDoux, 1999; Phelps and LeDoux, 2005; Wilensky et al., 2006; Duvarci and Pare, 2014; Sun et al., 2020). The LA, together with and the lateral nucleus of the central amygdala (CeL) are required for fear memory acquisition (Fanselow and LeDoux, 1999; Phelps and LeDoux, 2005). The medial nucleus of the central amygdala (CeM) is required for the expression of conditioned fear responses (Pascoe and Kapp, 1985; LeDoux et al., 1988; Wilensky et al., 2006; Ciocchi et al., 2010; for review see LeDoux, 2000). The CeM is also the main amygdala output structure to brainstem centers that mediate autonomic and behavioral aspects of fear, including freezing behavior (Hopkins and Holstege, 1978; Schwaber et al., 1982; Rizvi et al., 1991). The regulation of contextual fear memory has been attributed to the hippocampal-amygdala pathway (Rudy and O’Reilly, 1999; Rudy et al., 2004; Maren et al., 2013; Kim and Cho, 2020).

Both innate and learned fears are adaptive, defensive behaviors necessary for survival. However, maladaptive processing of fear memories can contribute to stress-related psychiatric disorders like post-traumatic stress-disorder (PTSD) and anxiety disorders. PTSD is characterized by hypervigilance, deficits in fear extinction, high reactivity to unpredictable vs predictable threat signals, and inability to properly discriminate between stimuli that predict danger vs stimuli that predict safety (Grillon et al., 2009; Jovanovic et al., 2009). Because OT has shown anxiolytic properties in animal (Bale et al., 2001; Missig et al., 2010; for review see Neumann and Slattery, 2016) and human studies (Lang et al., 2000; MacDonald and Feifel, 2014; for review see Janeček and Dabrowska, 2019), it has been extensively tested as a potential pharmacotherapeutic for these neuropsychiatric disorders.

However, despite promising results, the role of OT in the regulation of fear learning and extinction has proven more complex such as it is brain-site specific, sex-specific, and dependent on prior stress exposure. For example, OT was shown to reduce contextual fear expression in the central amygdala (CeA) (Viviani et al., 2011; Knobloch et al., 2012; Campbell-Smith et al., 2015) but the opposite effect was found in the medial prefrontal cortex (mPFC; Lahoud and Maroun, 2013; Brill-Maoz and Maroun, 2016) or the BLA (Lahoud and Maroun, 2013). In addition, OT has shown unique fear-modulating properties in different phases of fear learning, even within the same brain structure. For example, in the CeA, when manipulated before the fear acquisition phase, OT reduces contextual fear expression (Campbell-Smith et al., 2015), but increases (Lahoud and Maroun, 2013) or has no effect on fear expression when injected before fear recall (Campbell-Smith et al., 2015). Facilitating effects of OT on fear memory were observed in socially defeated mice, which demonstrated increased contextual fear responses in the presence of dominant intruders, an effect dependent on OTRs in the lateral septum (LS; Guzmán et al., 2013).

In contrast to contextual fear regulation, surprisingly fewer studies have investigated the effects of OT on the modulation of cued fear (Toth et al., 2012; Moaddab and Dabrowska, 2017; Terburg et al., 2018; Martinon et al., 2019). No reported studies to date have investigated the role of OTRs in the modulation of cued fear in the CeA or LA, both critical for cued fear acquisition. Even fewer studies have investigated the role of the endogenous OT system in the modulation of cued or contextual fear learning by investigating a recruitment of OT neurons during fear learning or using an OTR antagonist alone (Toth et al., 2012; Moaddab and Dabrowska, 2017). As a result, the role of endogenous OT in the regulation of fear still remains elusive.

**FIGURE 1:**
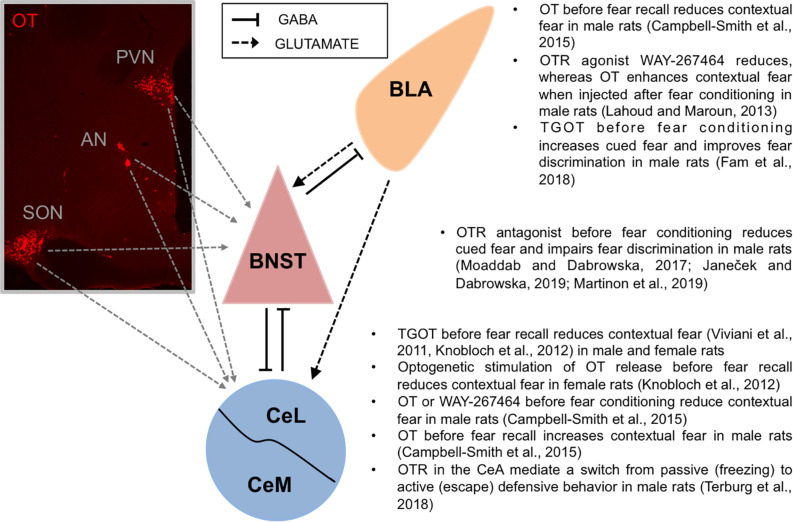
Oxytocin fosters accurate fear discrimination. **Left:** Oxytocin neurons produced in hypothalamic nuclei (PVN, AN, and SON) project to the extended amygdala: the BNST and the CeA (Dabrowska et al., 2011; Knobloch et al., 2012; Martinon et al., 2019). Mouse monoclonal anti-oxytocin antibody (1:7500, MAB5296, Millipore-Sigma) was used to visualize oxytocin neurons in the hypothalamus. **Right:** Accurate fear discrimination can be defined as an ability to discriminate and primarily respond to cues predicting imminent threats while showing diminished fear responses to un-signaled, diffuse, or distant threats. In the CeA, OTR activation has been shown to improve fear discrimination by reducing contextual fear responses. In the BNST, OTR neurotransmission has been shown to improve fear discrimination by increasing fear responses to discrete cues (cued fear) and by attenuating fear responses toward un-signaled threats (non-cued fear). Similarly, in the BLA, OTR activation significantly improved discrimination between responses to threat-predicting cue (paired with shock, CS+) vs safety-predicting cue (un-paired CS-), by potentiating fear responses to the CS+. AN, accessory nucleus of the hypothalamus; BLA, basolateral nucleus of the amygdala; BNST, bed nucleus of the stria terminalis; CeL, lateral part of central amygdala; CeM, medial part of central amygdala; OT, oxytocin; PVN, paraventricular nucleus of the hypothalamus; SON, supraoptic nucleus of the hypothalamus.

In this article, we review the effects of OT (or OT agonists) and OTR antagonists in the modulation of contextual and cued fear, primarily focusing on the effects in different amygdala nuclei and the bed nucleus of the stria terminalis (BNST). We highlight studies investigating the recruitment of endogenous OT neurons in the modulation of fear. With regard to behavioral effects of OT, we emphasize the ostensibly contrasting effects of OT in strengthening fear memory to predictable or signaled threats (cued fear) yet attenuating fear memory to unpredictable, diffuse threats (contextual fear and non-cued fear) (Missig et al., 2010; Ayers et al., 2011, 2016; Moaddab and Dabrowska, 2017; Janeček and Dabrowska, 2019; Martinon et al., 2019). Based on the fast-growing evidence, we propose that OT fosters accurate fear discrimination and adaptive defensive behaviors by increasing salience of relevant and imminent threats, yet reducing sustained fear responses to distant or un-signaled threats. Finally, based on the critical role of OT in the modulation of social behaviors, we also discuss the role of OT in the social transmission and social buffering of fear. With regard to the role of OT in enhancing the salience of both positive and negative social experiences (social salience hypothesis; for review see Shamay-Tsoory and Abu-Akel, 2016), we extend this idea by proposing that OT enhances the salience of environmental cues also in non-social contexts, and promotes active defensive behaviors (Figure 1). As OT effects on fear modulation are dose-dependent (Toth et al., 2012), we emphasize a wide range of doses used for investigating the behavioral studies, arguing that higher doses of OT might have inadvertently produced non-specific effects via vasopressin receptors (Chini and Manning, 2007; Manning et al., 2008). Finally, we also emphasize sex-specific effects of OT, when applicable.

## The Involvement of Endogenous Oxytocin System in the Modulation of Contextual and Cued Fear Conditioning

Several studies have shown that hypothalamic OT neurons are activated (measured as a c-fos expression) during fear-conditioning and fear recall (Zhu and Onaka, 2002; Hasan et al., 2019; Martinon et al., 2019). We have recently shown that cued and contextual fear conditioning activate hypothalamic neurons differently, such that contextual fear conditioning (un-signaled foot-shocks) activates OT neurons in the PVN, SON, and AN, whereas cued fear conditioning (foot-shocks signaled by a cue) activates OT neurons in the SON and AN, but not the PVN of male rats. Overall, contextual fear conditioning caused more robust activation of OT neurons than cued fear conditioning in all three hypothalamic nuclei, suggesting that OT neurons are activated more in the conditions of uncertainty (Martinon et al., 2019). Previously, contextual fear conditioning was shown to activate magnocellular OT neurons in the PVN and SON of male rats (Zhu and Onaka, 2002).

Stress-evoked OT release has been shown in the PVN (Nishioka et al., 1998; Neumann et al., 2000), LS (Zoicas et al., 2014) and the CeA (Ebner et al., 2005). However, the effects of fear learning on OT release are largely understudied. By using *in vivo* microdialysis in freely moving male rats, we have shown that cued fear-conditioning (foot-shocks signaled by a cue) evokes OT release in the dorsolateral BNST (BNST_*DL*_) (Martinon et al., 2019). This increase was specific to cued fear acquisition because un-signaled foot-shocks or forced swim stress failed to alter OT content in BNST_*DL*_ microdialysates (Martinon et al., 2019). These results suggest that OT is specifically released in the BNST_*DL*_ during cued fear learning.

A novel method based on virus-delivered genetic activity-induced tagging of cell ensembles (vGATE) was applied to label fear-experience-activated OT neurons in the female rat hypothalamus. Here, a *c-fos* promoter fragment was modified to allow a sustained expression for permanent tagging of c-fos-expressing neurons. This allowed the demonstration that OT neurons in the PVN and SON are activated during contextual fear-conditioning in a specific context A and a similar fraction of these neurons is re-activated after exposure to the same context A on the next day. When the same rats were exposed to a different context B, a significantly higher number of activated OT neurons was found, indicating that a new population of OT neurons was recruited (Hasan et al., 2019). These fear-activated OT neurons were found projecting specifically to the CeL. Notably, optogenetic activation of the CeL-projecting OT neurons reduced contextual fear, whereas their silencing impaired context-specific fear extinction (Hasan et al., 2019). These results highlight the important role of OT in the CeA in attenuating contextual fear memory, which has been demonstrated by numerous studies (Viviani et al., 2011; Knobloch et al., 2012; Campbell-Smith et al., 2015).

In contrast to the results above, systemic administration of OTR antagonist, atosiban, was shown to reduce fear consolidation, suggesting that endogenous OT facilitates the consolidation of long-term contextual fear memories in male rats (Rasie Abdullahi et al., 2018). Atosiban, administered at different doses (1, 10, 100 or 1000 μg/kg) immediately after a fear conditioning session (consolidation phase), disrupted fear expression (tested 48 h later) in a dose-dependent manner (with 10 and 100 μg/kg doses being the most effective). Although these results disagree with the effects of OT in the CeA attenuating contextual fear (Hasan et al., 2019), systemic administration of OTR antagonist might have potentially affected other brain regions, including LA and/or BLA, essential for consolidation of fear memories. In addition, as described in the following section, OT has shown some contrasting effects on contextual fear, dependent on the timing of the administration and brain region involved.

## The Effects of Oxytocin in the Regulation of Contextual Fear

### Central Nucleus of the Amygdala

By modulating the activity of the amygdala, OT plays an essential role in the regulation of fear. The majority of studies to date show that OT in the CeA reduces expression of contextual fear in rodents (Viviani et al., 2011; Knobloch et al., 2012; Campbell-Smith et al., 2015) but see Lahoud and Maroun (2013). Following 2 days of contextual fear conditioning, male and female rats received bilateral intra-CeA injections of the specific and potent OTR agonist, [Thr4,Gly7]-oxytocin (TGOT; 7 ng), or vehicle. When injected before the fear recall test, TGOT-treated rats showed decreased freezing responses to the context, suggesting accelerated extinction. During *in vitro* patch-clamp electrophysiological recordings, TGOT selectively excited and facilitated firing of interneurons located in the CeL. In addition, in retrogradely labeled CeA projection neurons, TGOT increased inhibitory synaptic transmission (measured as a frequency of spontaneous inhibitory postsynaptic currents, sIPSC) selectively in CeM neurons that project to the periaqueductal gray (PAG, responsible for freezing behavior), but not CeM neurons that project to dorsal vagal nuclei (DVC, responsible for cardiovascular response). These results, observed in both male and female rats, suggest that TGOT accelerated fear extinction via OTR-expressing CeL neurons that inhibit the PAG-projecting CeM neurons (Viviani et al., 2011).

These results were confirmed by a later study showing that optogenetic stimulation of OT release activates OTR-expressing CeL neurons and reduces contextual fear. Here, female rats were injected with a recombinant adeno-associated virus (rAAV) driving the expression of channelrhodopsin 2 (ChR2) fused with mCherry, under the OT promoter, to all three hypothalamic nuclei (PVN, SON, and AN). During cell-attached recordings in the CeA slices, blue light increased action potential frequency of one-third of CeL neurons, which was blocked by pre-incubation with OTR antagonist d(CH2)5-Tyr(Me)-[Orn8]-vasotocin (OTA, 1 μM), suggesting a direct excitatory effect of OT. As a result, an exposure to blue light also caused an increase in sIPSC frequency in CeM neurons, which was also reversed by OTA (Knobloch et al., 2012), confirming previous results by Viviani et al. (2011). In the Knobloch study, virgin female rats were also tested for contextual fear recall after 2 days of contextual fear-conditioning training. Optogenetic stimulation of OT release in the CeA prior to testing reduced freezing responses, an effect abolished by OTA (Knobloch et al., 2012). Both of these studies suggest that OT (either via injection or optogenetically stimulated release) primarily activates CeL neurons, which in turn increases GABA-ergic inhibition in PAG-projecting CeM output neurons, leading to the attenuation of contextual fear responses (for details on the OT-modulated neurocircuitry, see Beyeler and Dabrowska, 2020). In contrast to the study by Knobloch and colleagues (2012) in female rats, infusion of OT at various doses (0.6, 3, 15, and 75 ng) in the CeA of male rats before the first recall test resulted in higher levels of freezing during the test (Campbell-Smith et al., 2015). However, non-peptide OTR agonist WAY-267464 (3 μg), synthetic OT (10 ng), or saline injected into the CeA after fear recall test resulted in no significant differences in freezing responses between groups in male rats (Lahoud and Maroun, 2013). In the latter study, when OTR agonists, TGOT (7 ng) or WAY-267464 (as above), were infused into the CeA before fear conditioning (fear acquisition), both significantly reduced contextual fear recall measured on the next day. Both agonists facilitated subsequent extinction, whereas synthetic OT infused into the CeA had no effect (Lahoud and Maroun, 2013). Direct infusion of OT (75 ng) into the CeA before fear acquisition (two-shock exposure) also impaired contextual fear expression measured on the next day (Campbell-Smith et al., 2015).

Therefore, sex-specific effects of OT might, at least in part, contribute to the different behavioral outcomes as the contextual fear-reducing effects of OT have been consistently shown in female rats (Viviani et al., 2011; Knobloch et al., 2012), but mixed results were obtained in males (Viviani et al., 2011; Lahoud and Maroun, 2013; Campbell-Smith et al., 2015). In addition, some of the discrepancies between the OT effects might be due to the different doses used (75 ng vs 10 ng), considering that OT effects on fear are dose-dependent (Toth et al., 2012). Additionally, as OT has a relatively high affinity for vasopressin 1A receptors (AV1R) (Chini and Manning, 2007; Manning et al., 2008), this raises a possibility of non-specific behavioral effects via V1AR when using higher OT doses. Hence, it is critical to demonstrate OTR-specific effects by using a selective OTR antagonist.

### Basolateral Nucleus of the Amygdala

OT has been shown to modulate BLA activity during contextual fear learning to improve long-term extinction in male rats. OT infused into the BLA (doses 0.6, 3, 15, and 75 ng) before extinction training (fear recall test) impaired the expression of contextual fear. During the first fear recall test, rats that received OT (all doses) showed decreased freezing levels in comparison with vehicle-treated rats, and similar effects were observed during the second fear recall test (except for the lowest 0.6 ng dose, which had no effect). This facilitation of extinction was blocked by the co-administration of selective OTR antagonist desGly-NH2-d(CH2)5[D-Tyr2,Thr4]OVT (OTA, 15 ng), demonstrating OTR-mediated effects (Campbell-Smith et al., 2015). In a different study, male rats received injections of synthetic OT (0.01 μg), OTR agonist WAY-267464 (3 μg), TGOT (7 ng) or vehicle into the BLA after contextual fear conditioning. Here, OT- and WAY-267464-treated rats showed significant differences in comparison to vehicle- and TGOT-treated rats. Whereas WAY-267464-treated rats showed significantly lower freezing levels in comparison with the other groups, OT-treated rats showed enhanced freezing levels, suggesting increased consolidation of fear. Additionally, TGOT had no effect (Lahoud and Maroun, 2013).

These results indicate that in the BLA, synthetic OT improves extinction of contextual fear when manipulated before fear recall test but impairs extinction when manipulated after conditioning. Notably, injection of WAY-267464 in the BLA after conditioning accelerates fear extinction. However, this behavioral effect might be partly due to V1AR modulation, as WAY-267464 demonstrated high affinity for V1AR (Hicks et al., 2012; Jurek and Neumann, 2018).

Interestingly, although the regulation of contextual fear memory has been attributed to the hippocampal-amygdala pathway (Rudy and O’Reilly, 1999; Rudy et al., 2004; Maren et al., 2013), and OT in the hippocampus was shown to modulate social memory (Cilz et al., 2018), no studies to date have investigated the role of OT in contextual fear in this brain region.

## The Role of OT in the Regulation of Cued Fear Conditioning

### The Basolateral and the Central Amygdala

OT has been shown to play an important role in the BLA-CeA-mediated switch from passive (freezing) to active (escape) defensive behaviors in rodents. The exposure to an imminent, yet escapable, threat selectively activated the BLA projections to a group of OT-sensitive neurons in the CeL (Terburg et al., 2018). Here, male rats were exposed to a threat and escape task in a two-way shuttle box in which threats could be distant, imminent, or inescapable. Rats were presented with tones (the CS) that varied in frequency, intensity, and length, and co-terminated with a foot-shock. Tones of higher frequency announced higher imminence of the aversive event. Rats learned to escape by moving to the other compartment of the box during the presentation of the tone. Failure to escape resulted in the foot-shock. Before the threat and escape task, rats received a direct injection of OTR agonist, TGOT (7 ng), into the CeL, which increased the escape performance and decreased freezing responses to imminent but not distant threats. Additionally, application of OTA (42 ng) into the CeL decreased escape performance to imminent threat while increased freezing responses to both distant and imminent threats in comparison with vehicle-treated rats. These results suggest that blocking OTR transmission in the CeA reverses the defensive behavior from an adaptive to a maladaptive state, in which rats do not escape when encountered with an imminent threat and instead, begin freezing to threats which do not pose an immediate danger. The BLA input was demonstrated to be essential for the OTR-mediated selection and execution of the active escape responses to an immediate threat. When a stimulating electrode was placed in the BLA, less current was needed to evoke an action potential in OT-sensitive neurons in the CeL slice (prepared immediately after threat and escape learning session) (Terburg et al., 2018).

### The Bed Nucleus of the Stria Terminalis

In contrast to the CeA, which has been shown to mediate fear responses to short, discrete cues (cued fear), the BNST primarily mediates anxiety-like responses, as well as fear responses to unpredictable, diffuse, or un-signaled threats (Walker and Davis, 2008; Davis et al., 2010; Goode et al., 2019). The BNST might also inhibit cued fear (Meloni, 2006). Yet, using fear potentiated-startle (FPS), we demonstrated that OTR neurotransmission in the BNST_*DL*_ facilitates acquisition of cued fear (Moaddab and Dabrowska, 2017), and it reduces fear responses to un-signaled, diffuse threats (Janeček and Dabrowska, 2019; Martinon et al., 2019). In the FPS experiment, an acoustic startle reflex (which occurs <200 msec after a white noise burst) is potentiated during an exposure to a CS (e.g., light) that has been previously paired with a foot-shock during the fear conditioning session. In the FPS recall test, animals are presented with startle-eliciting bursts in the presence or absence of the CS (mixed in a pseudorandom order) (Davis et al., 1993; Davis, 2001; Walker and Davis, 2002). Both cued fear and non-cued fear can be measured during FPS. Whereas cued fear is the startle potentiation measured during cue presentations, non-cued fear is measured as the startle potentiation observed in between the cue presentations (Missig et al., 2010; Ayers et al., 2011; Moaddab and Dabrowska, 2017; Janeček and Dabrowska, 2019; Martinon et al., 2019). However, as the latter response does not occur until after the cues are being presented, it can be used as a proxy of how well an animal discriminates between the presence and the absence of the cue. In the study, we injected OT (100 ng), OTR antagonist d(CH2)51, Tyr(Me)2, Thr4, Orn8, des-Gly-NH29)-vasotocin (OTA; 200 ng), or vehicle (ACSF) directly into the BNST_*DL*_ of male rats before or after fear-conditioning (acquisition and consolidation, respectively). When administered before fear conditioning, OTA-treated rats showed a significant reduction of cued fear measured on the next day in comparison to vehicle-treated rats. We also observed a consistent trend in an OTA-induced increase in the non-cued fear. No significant differences were found when OT, OTA, or vehicle were injected after the fear conditioning session (consolidation phase) (Moaddab and Dabrowska, 2017). These results indicate that OTR neurotransmission in the BNST_*DL*_ facilitates the acquisition of fear to a discrete cue. We later confirmed the recruitment of endogenous OT in the acquisition of cued fear memory with a microdialysis study showing that cued-fear conditioning indeed evokes OT release in the BNST_*DL*_ (Martinon et al., 2019).

However, how does one explain that OTR transmission in the BNST promotes cued fear, whereas the BNST activation produces opposite effects? Our recent electrophysiological findings in male rats show that OTR in the BNST_*DL*_ might facilitate cued fear by inhibiting the BNST (Francesconi et al., 2020). Here, we showed that in the BNST_*DL*_, OT selectively excites and increases firing rate of Type I interneurons, which potentiates inhibitory synaptic transmission (frequency of sIPSCs) selectively in Type II projection neurons. Therefore, similarly to the OT effects in the CeA (Viviani et al., 2011; Terburg et al., 2018), there are also two classes of OT-responsive neurons in the BNST_*DL*_: a class of interneurons directly excited by OT and a class of projections neurons indirectly inhibited by OT. We also used retrograde fluorescent labeling to record specifically from CeA-projecting BNST_*DL*_ neurons, which we then identified as Type II. Therefore, our results suggest that OTRs in the BNST_*DL*_ promote cued fear by inhibiting the CeA-projecting BNST_*DL*_ neurons (Francesconi et al., 2020). As the BNST-CeA projection is GABA-ergic, OT might disinhibit the CeA and therefore increase cued fear.

## Other Brain Regions

When OT (10 ng) or non-peptide OTR agonist WAY-267464 (3 μg) were infused after the first fear recall test into the infralimbic cortex (IL), part of the medial prefrontal cortex (mPFC), male rats showed significant reduction in freezing behavior and facilitation of subsequent fear extinction (Lahoud and Maroun, 2013). OTR-mediated facilitation of fear extinction was later confirmed with TGOT administration into the mPFC (7 ng administered before the second recall session), which led to reduced freezing levels in comparison with OTA-(74.8 ng) or vehicle-treated rats (Brill-Maoz and Maroun, 2016). Interestingly, TGOT (7 ng) infusion into the IL had no effect on fear extinction in juvenile male rats (Kritman et al., 2017). These results suggest that OTRs in the mPFC might facilitate fear extinction learning in adult rats but not during adolescence when PFC GABA-ergic circuits are still largely under neurodevelopment (Caballero et al., 2016).

## The Effects of Intracerebroventricular, Systemic and Intranasal Administration of OT on the Regulation of Cued Fear

Intracerebroventricular (ICV) injection of OT was shown to facilitate cued fear extinction whereas OTA (desGly-NH2,d(CH2)5[Tyr(Me)2,Thr4]OVT) impaired the extinction when infused before fear conditioning in male rats (Toth et al., 2012). During fear recall sessions over the following 2 days, OT-treated rats (0.1 and 1.0 μg) showed significantly lower freezing whereas OTA-treated rats (0.75 μg) showed higher freezing responses in comparison with controls. The authors also determined the effects of OT on cued fear when administered before fear recall in both rats and mice. In rats, OT (0.1 and 1.0 μg) increased freezing response compared to vehicle in an OTR-dependent manner, whereas in mice, OT showed a dose-dependent effect such that a lower OT dose (0.1 μg) increased freezing whereas a higher dose (0.5 μg) decreased freezing responses compared with controls (Toth et al., 2012). In contrast, no significant effect of OT was found on cued fear recall when different doses (0.002, 0.02, 0.1, 0.2, and 2 μg) were administered ICV before fear-potentiated startle test (Ayers et al., 2011). Overall, these results indicate that ICV OT modulates cued fear extinction in a dose- and time-dependent fashion, depending on when in the learning process OT was administered and what behavioral outcome was measured (freezing vs startle).

Intranasal OT reduced chronic stress-induced deficits in fear extinction in male rats. Animals underwent a protocol of chronic stress (restrained stress, forced swim, and diethyl ethyl vapor anesthesia, all in the same day) (Wang S.-C. et al., 2018). Here, intranasal OT (1 μg) or vehicle was administered before cued fear conditioning in previously stressed and non-stressed rats. Two days later, stressed rats that received vehicle showed enhanced freezing levels during fear-recall session in comparison to non-stressed rats as well as in comparison to stressed rats that received OT.

Based on the widespread OTR expression and brain region-specific role of OT, caution needs to be applied when interpreting results based on ICV, and especially based on systemic or intranasal OT administration. Although OT administered intranasally has been shown to modulate amygdala activity in humans (Kreuder et al., 2020) and increase OT levels in the amygdala in rodents (Neumann et al., 2013), other brain regions might also be involved in the behavioral OT effects.

## The Role of Oxytocin in the Regulation of Fear Discrimination

After cued fear conditioning, fear discrimination can be defined as an ability to distinguish and primarily respond to cues predicting a learned aversive event versus cues that have no or little prognostic value of a threat (Janeček and Dabrowska, 2019; Martinon et al., 2019). The un-predictable or non-prognostic cues can be neutral (no association with either threat or safety) or they can signal safety. Fear discrimination can be also defined as an ability to discriminate between signaled vs un-signaled threats (signal or cue predicts threat, whereas lack of signal represents threat apprehension) (Overmier et al., 1971; Goode et al., 2019). Accurate fear discrimination during confrontation with an immediate threat or threat-predicting cue is an adaptive response which promotes survival. On the other hand, threat apprehension and/or inability to attenuate fear responses toward neutral or safe stimuli leads to fear generalization. After Pavlovian fear conditioning, cued fear is measured as an increase in freezing behavior or potentiation of a startle response during presentation of a cue (CS+), which was previously paired with an aversive event (i.e., foot-shock). In order to measure fear discrimination, an additional cue is introduced, which is not paired with foot-shock (CS-), and fear responses to both CS+ vs CS− are measured during fear recall (Duvarci et al., 2009). Fear discrimination between signaled vs un-signaled threats can be measured during FPS, where in addition to measuring startle responses during cue presentations, one can also measure startle potentiation observed between the cue presentations. This phenomenon, so called non-cued fear (Moaddab and Dabrowska, 2017; Janeček and Dabrowska, 2019; Martinon et al., 2019) or background anxiety (Missig et al., 2010; Ayers et al., 2011, 2016) does not occur until after the cue presentations during FPS recall and therefore can be used as a proxy of how well an animal discriminates between the presence and the absence of the cue. Here, fear discrimination can be measured as the proportion between cued fear and non-cued fear (Janeček and Dabrowska, 2019; Martinon et al., 2019).

Subcutaneous OT administration in male rats has been shown to reduce background anxiety (or non-cued fear) measured in the FPS (Missig et al., 2010). Here, rats were fear conditioned and tested for FPS 96 h later. During fear recall, they were tested for acoustic startle responses in the presence or absence of the cue, presented in a pseudorandom order. OT was administered (0.001, 0.1, or 1.0 μg/kg) before acquisition, consolidation or expression of fear. Injections of OT before fear conditioning had no effect on the FPS acquisition or consolidation. However, although OT administered before fear recall had no effect on cued fear expression, it significantly reduced non-cued fear at the 0.1 μg/kg dose (Missig et al., 2010). These results suggest that OT attenuates fear responses to un-signaled, diffuse threats and as such might improve fear discrimination. This FPS study was later replicated, confirming that systemic administration of OT (0.01 or 0.1 μg/kg) reduces non-cued fear, with no effect on cued fear. Surprisingly, ICV administration of OT (at doses ranging from 0.002 to 20 μg) had no effect on any component of the FPS (Ayers et al., 2011). However, when rats were grouped into low and high startle responders (based on pre-fear baseline levels), ICV OT was shown to reduce the non-cued fear in rats with low baseline startle responses (Ayers et al., 2016).

In search for potential brain sites where OT might act to improve fear discrimination, Fam et al. (2018) showed that OTR transmission in the BLA enhances acquisition of fear to the CS+. Here, male rats were trained using discriminatory fear conditioning with two different auditory cues: one paired (CS+) and another un-paired (CS−) with a foot-shock. Immediately before two conditioning sessions, rats received either infusion of OTR agonist, TGOT (7 ng), or vehicle directly to the BLA. During fear recall test, as expected, the overall freezing responses were significantly higher to CS+ than to CS−. However, the observed difference was significantly greater in the TGOT group with the response primarily driven by significantly greater freezing to CS+. Hence, OTR activation in the BLA resulted in the facilitation of cued fear acquisition and an improvement of fear discrimination (Fam et al., 2018). In another experiment, TGOT also strengthened the discrimination between reinforced compound CSX+ and a non-reinforced compound CSX−, when additional cue X was added to both existing cues during conditioning. The difference in freezing between CSX+ and CSX− was significantly higher in TGOT-treated rats than control rats, whereas control rats showed poor discrimination abilities (Fam et al., 2018).

BLA inputs to a group of OT-sensitive neurons in the CeL were also shown to improve fear discrimination by facilitating a switch from passive (freezing) to active (escape) defensive behavior and permitting rapid defensive behavior in rats (Terburg et al., 2018). Here, TGOT injected into the CeL increased the escape performance to imminent threats and decreased freezing responses to imminent but not distant threats. Application of OTA decreased escape performance to imminent threat while increasing freezing responses to both distant and imminent threats in comparison with vehicle-treated rats.

The BLA sends excitatory inputs also to the BNST (Gungor and Pare, 2016) and growing evidence suggests that the BNST plays a major role in the modulation of fear discrimination (Duvarci et al., 2009; Goode et al., 2019, 2020; Janeček and Dabrowska, 2019; Martinon et al., 2019). Male rats with BNST lesions were trained in a discriminatory auditory fear conditioning paradigm (paired CS+ vs un-paired CS−) and showed significantly improved fear discrimination during fear recall test, such that they responded primarily to the CS+ while showing significantly reduced responses to CS− (Duvarci et al., 2009). Recently, we demonstrated that OTR neurotransmission in the BNST_*DL*_ facilitates the acquisition of cued fear while attenuating fear responses to un-signaled, diffuse threats, overall improving fear discrimination (Janeček and Dabrowska, 2019; Martinon et al., 2019). Using FPS, we showed that OTA (200 ng) injected into the BNST_*DL*_ before fear conditioning significantly reduced cued fear measured on the next day. In addition, OTA showed a trend to increase non-cued fear (Moaddab and Dabrowska, 2017). Therefore, we also calculated discrimination index for individual rats (percentage change of cued fear over percentage change of non-cued fear) and showed that blocking OTRs in the BNST_*DL*_ completely disabled fear discrimination (Janeček and Dabrowska, 2019; Martinon et al., 2019). Our recent electrophysiological study suggests that OTR neurotransmission in the BNST_*DL*_ facilitates fear discrimination by inhibiting BNST output (Francesconi et al., 2020).

Overall, OTR neurotransmission improves fear discrimination by increasing fear responses toward imminent threats or cues that predict imminent threats (cued fear, fear to CS+), or/and by attenuating fear responses toward un-signaled, un-predictable or distant threats or safety signals (CS−). Therefore, OT appears to promote adaptive fear responses by increasing the salience of relevant threat-signaling cues and reducing fear responses to diffuse or distant threats that do not require an immediate reaction (Figure 1).

## Oxytocin and the Social Transmission of Fear

OT is well-known for its role in mediating affiliate and prosocial behaviors (for review see Neumann and Slattery, 2016) and modulating cooperation and conflict among humans during intergroup relationships (for review see Shamay-Tsoory and Abu-Akel, 2016). Emerging evidence suggests that OT is also involved in the regulation of emotional discrimination, social buffering of fear, and social transmission of emotions, including fear (Guzmán et al., 2013; Ferretti et al., 2019; Hirota et al., 2020).

OTR neurotransmission in the lateral septum (LS) was shown to attenuate social fear conditioning in male mice (Zoicas et al., 2014). Here, mice placed in a chamber next to a cage with an unfamiliar conspecific were either allowed to freely explore the conspecific (unconditioned group) or they received a foot-shock each time they made direct contact with the conspecific mouse (socially fear-conditioned group). On the next day, both groups received ICV injections of either OT (0.1 or 0.5 μg) or vehicle before fear recall test. Vehicle-injected mice that were socially conditioned showed reduced social investigation in comparison with unconditioned mice. In contrast, both doses of OT increased social investigation in socially conditioned mice to the levels of the unconditioned mice, suggesting that OT abolished social fear expression. Next, selective OTR antagonist, desGly-NH2,d(CH2)5[Tyr(Me)2,Thr4]OVT (OTA; 2 μg) was used before fear recall with or without OT or vehicle. Both unconditioned mice that received OTA/vehicle and conditioned mice that received OTA/OT showed reduced social investigation in comparison with vehicle-treated, unconditioned mice (Zoicas et al., 2014). These results suggest that OTR transmission in the LS regulates social investigation and counteracts the effects of social fear conditioning on social behavior in mice.

OTRs in the LS were also found crucial for strengthening fear memories associated with a negative social experience, such as acute social defeat (Guzmán et al., 2013). Here, the role of OTRs in contextual fear was investigated in male mice with adeno-associated virus (rAAV)-mediated OTR knockdown or overexpression in the LS. Although neither OTR knockdown nor overexpression resulted in differences in contextual fear in control mice, it impaired and enhanced contextual fear, respectively, in socially defeated mice subjected to the dominant intruder (Guzmán et al., 2013). These results demonstrate that OTR neurotransmission in the LS potentiates fear memories associated with a previously experienced social threatening context.

On the other hand, OTR neurotransmission has been shown to attenuate fear response when associated with a rewarding experience, such as pair bonding (Hirota et al., 2020). Here, male voles were first cohabitated with an unfamiliar naïve female. Next, they received ICV injection of selective OTR antagonist, desGly-NH2, d(CH2)5[Tyr(Me)2,Thr4]OVT (OTA; 0.25 μg), or vehicle before conditioning training in a passive avoidance test. Pair-bonded voles, but not unpaired controls, injected with OTA, showed an increase in fear memory manifested by significantly delayed latency to enter the dark box previously associated with foot-shock. These results suggest that OTR signaling underlies the buffering effect of pair bonding on fear learning.

OTR transmission has been also shown to play a role in consolation behavior in prairie voles as they displayed more grooming behavior toward a familiar conspecific that had experienced a stressful event (five foot-shocks). Both female and male observers showed increased duration and reduced latency of allogrooming to the stressed demonstrator vs non-stressed voles. Exposure to the stressed conspecific increased activity (measured as c-fos expression) in the anterior cingulate cortex of the observer. Notably, OTR antagonist (peptidergic ornithine vasotocin analog desGly–NH2,d(CH2) 5 [Tyr(Me)2,Thr4]OVT, OTA; 2.5 ng) infused ICV or to the cingulate cortex before the consolation, prevented the partner-directed grooming response (Burkett et al., 2016). These results suggest that OTR neurotransmission in the anterior cingulate cortex is fundamental for consolation behavior toward stressed familiar conspecifics in voles. Notably, consoling voles also displayed fear-like behaviors after observing their partners in distress.

This form of socially transmitted fear was also observed in mice. When a familiar conspecific was stressed by foot-shocks, the observer mouse became fearful, a phenomenon called observational fear (Pisansky et al., 2017). Here, female observer mice showed similar observational fear toward both familiar and unfamiliar conspecifics. In contrast, male observers demonstrated significantly less observational fear toward unfamiliar conspecifics compared to familiar ones. However, intranasal administration of OT prior to fear conditioning of an unfamiliar demonstrator resulted in significant increase in freezing (compared with vehicle-treated), whereas no effect of OT treatment was found when observing familiar males. Notably, systemic administration of OTR antagonist L-368,899 hydrochloride (5 or 10 mg/kg, IP) reduced observational fear in familiar male mice (compared to vehicle-treated). Finally, the authors utilized designer receptors exclusively activated by designer drugs (DREADDs) to chemogenetically activate OT neurons in the PVN before demonstrator conditioning, which resulted in significant enhancement of freezing responses toward unfamiliar demonstrators (Pisansky et al., 2017). Overall, these results suggest that OTR signaling underlies social transmission of fear between familiar conspecifics, whereas activation of the OT system can increase observational fear also toward unfamiliar conspecifics in male mice. Social transmission of fear between distressed conspecifics might serve as warning system of impeding threat.

OTR signaling has been also implicated in emotional discrimination. CeA-projecting OT neurons from the PVN were shown to mediate discrimination between behaviors of different emotional valence (Ferretti et al., 2019). Here, two demonstrators were first exposed to a tone (neutral group) or pairings of tone and foot-shock (fearful group). Both demonstrators were returned to the same context in the presence of an unfamiliar observer mouse on the next day where they were exposed to the tone followed by 2 more minutes without the tone. Both female and male observers increased sniffing toward the fearful mouse compared with the neutral control after the tone presentation. An inverse correlation was found between the time the demonstrator was freezing and the time of the observer sniffing, which suggests that freezing behavior might influence the observer discrimination. However, no correlation was found between demonstrator freezing and observer sniffing after tone presentation, indicating that demonstrator freezing did not affect the discriminatory behavior in the observer (Ferretti et al., 2019). Interestingly, chemogenetic inhibition of the CeA-projecting OT neurons from the PVN prevented the ability of male and female mice to discriminate between fearful vs neutral states in conspecifics. In contrast, selective inhibition of PVN neurons projecting to nucleus accumbens (NAcc), mPFC, and CA2 region of hippocampus did not affect these discriminative abilities. Overall, these results demonstrate that OTR transmission in the CeA is crucial for emotion discrimination in female and male mice.

OTR signaling in the mPFC was also shown to play a critical role in social buffering of fear in rats (Brill-Maoz and Maroun, 2016). After contextual fear conditioning, male rats were tested in two fear recall sessions either alone (single group) or in pairs (paired group). Paired rats showed lower freezing levels than rats that were alone, indicating that fear extinction in pairs is accelerated. Then, TGOT (7 ng), OTR antagonist desGly-NH2,d(CH2)5[D-Tyr2,Thr4]OVT (OTA; 74.8 ng) or vehicle was injected into the mPFC before the second fear recall session in all groups. In both groups, TGOT-treated rats showed significantly reduced freezing levels in comparison with other groups. However, only in the paired group, OTA-treated rats showed significantly higher freezing than the other groups (Brill-Maoz and Maroun, 2016), suggesting that socially facilitated fear extinction is mediated by OTR neurotransmission in the mPFC.

## Concluding Remarks

Oxytocin neurons are recruited during fear memory formation and release OT into the CeA and the BNST, where OT has been shown to reduce contextual fear and increase cued fear, respectively. These ostensibly contrasting behavioral effects support growing evidence that OT fosters adaptive fear discrimination by reducing sustained fear responses and anxiety-like behaviors yet strengthening fear responses to relevant and predictable threats. Recent studies support this concept and show that OTR transmission in the CeA mediates a switch from passive freezing to active escape behaviors in confrontation with an imminent, yet escapable threat but reduces fear responses to distant or diffuse threats. Therefore, OT appears to increase salience of relevant threat-signaling environmental cues and promote active defensive behaviors. OT has been also shown to improve emotional discrimination between conspecifics, and OTR signaling underlies the social transmission of fear (observational fear), social buffering of fear, and consolation behaviors toward distressed conspecifics. As OT can strengthen fear memories associated with previously experienced threatening social or environmental stimuli, it can serve as a warning signal against impeding social and environmental threats. Here, we extend the social salience hypothesis and propose that in addition to OT enhancing salience of both positive and negative social experiences, it can also increase salience of relevant environmental threats and promote survival also outside of direct social context.

## Financial Disclosures

JD reports submission of a provisional patent application entitled: Method and System for Testing for Stress-related Mental Disorders, Susceptibility, Disease Progression, Disease Modulation, and Treatment Efficacy (#62/673447).

## Author Contributions

VO-P and JD wrote the manuscript. Both authors contributed to the article and approved the submitted version.

## Conflict of Interest

The authors declare that the research was conducted in the absence of any commercial or financial relationships that could be construed as a potential conflict of interest.
